# Pathophysiologic Study of the Changes of Blood Flow in Portal Vein and Hepatic Artery and the Role of Administration of Regadenoson in Post-hepatectomy Liver Failure

**DOI:** 10.7759/cureus.107716

**Published:** 2026-04-25

**Authors:** Dimitrios Massaras, Zoi Masourou, Grigorios Psarras, Eirini Pantiora, Kleoniki C Kordeni, Maria Massara, Elissaios A Kontis, Antonios Vezakis, Kassiani Theodoraki, Georgios P Fragulidis

**Affiliations:** 1 Surgery, Metaxa Anticancer Hospital, Piraeus, GRC; 2 Anesthesiology, Aretaieion University Hospital, National and Kapodistrian University of Athens School of Medicine, Athens, GRC; 3 Radiology, Aretaieion University Hospital, National and Kapodistrian University of Athens School of Medicine, Athens, GRC; 4 Surgery, Aretaieion University Hospital, National and Kapodistrian University of Athens School of Medicine, Athens, GRC; 5 Radiology, Metaxa Anticancer Hospital, Piraeus, GRC

**Keywords:** adenosine, liver failure, portocaval shunt, posthepatectomy, regadenoson

## Abstract

Backround

Posthepatectomy liver failure syndrome is a well-described complication after extended hepatectomies, which contributes significantly to the perioperative morbidity and mortality of the patient. The major pathophysiological basis of this syndrome is the hepatic arterial buffer response (HABR), which reflects the hemodynamic changes that occur in the liver after a hepatectomy. As the portal vein is unable to adjust blood flow to the diminished hepatic parenchyma, the liver establishes a unique mechanism that leads to a decrease in the flow of hepatic artery via vasoconstriction. There are surgical techniques that are already applied to reverse this phenomenon, but they are still considered risky, as they carry high perioperative mortality. On the other hand, pharmacological modulation of the hepatic vasculature can reverse the HABR phenomenon and contribute to the treatment of post-hepatectomy liver failure (PHLF) syndrome. In our experimental research, we studied the administration of regadenoson, an A_2A_ adenosine agonist, which directly reverses the vasoconstriction of the hepatic artery, showing superior results in the modulation of hepatic vasculature, compared to a portocaval shunt.

Methods

We designed a prospective experimental cohort study comprising animals that underwent extended left hepatectomy (70%) and portocaval shunt to avoid bowel ischemia after the Pringle maneuver. Regadenoson was administered intra-arterially in the hepatic artery. Variations of liver blood flow were measured with ultrasound Doppler equipment.

Results

The results of our study have demonstrated that direct administration of regadenoson increased the hepatic artery velocity by reversing the HABR and by creating subsequent vasodilation of the hepatic artery. Additionally, the results of administration of regadenoson in comparison to portocaval (PC) shunt were superior and could potentially reduce the incidence of PHLF.

Conclusion

Regadenoson significantly enhanced hepatic arterial perfusion and may be associated with a reduced risk of developing PHLF syndrome compared to surgical modulation alone in the porcine model. These results position regadenoson as a promising therapeutic agent for optimizing liver perfusion in cases where portal venous flow is impaired.

## Introduction

The evolution of hepatic surgery has allowed the successful treatment of more challenging primary and metastatic neoplasms of the liver. However, despite great improvements in outcomes after major liver resections due to refinements in operative technique and advances in critical care, post-hepatectomy liver failure (PHLF) remains one of the most serious complications of major liver resection, and occurs in up to 10% of these cases [1, 2]. Posthepatectomy liver failure is a feared complication after extended hepatic resections and a major cause of perioperative mortality, with a percentage of 30% [3]. The aetiological factor of this fatal syndrome is multifactorial, and the pathophysiological basis is a complicated cascade concerning the vasculature, size, and quality of the liver remnant. One of the cornerstone events in the pathophysiology of PHLF is the increased blood flow to the liver remnant - portal overflow [4]. As the portal vein is unable to adjust the blood flow to the diminished hepatic parenchyma, the liver establishes a unique mechanism that leads to a diminished flow of the hepatic artery through arterial vasoconstriction. This mechanism is called the hepatic arterial buffer response (HABR) and is one of the main etiological factors of the post-hepatectomy liver failure syndrome [5].

As we come closer to understanding this complex and fatal syndrome, many surgical and pharmacological methods have already been introduced in practice to deal with PHLF. Management of this syndrome has led to the development of various surgical techniques with the aim of reversing it and decreasing the flow and the pressure of the portal vein, such as portocaval shunt (PCS), etc. [6-8]. Additionally, there are several medical substances that are being studied as a potential alternative method to increase the hepatic artery flow [9]. Surgical techniques that modulate hepatic flow, such as splenectomy, splenic artery ligation, and portocaval shunt, are still questionable in terms of efficacy and potential benefit to the patient. On the contrary, pharmacological modulation is a promising possibility but needs further experimental results.

The primary objective of this study is to compare regadenoson administration and PCS by evaluating changes in portal vein and hepatic artery velocities following extended left hepatectomy, and to determine whether intra-arterial regadenoson more effectively modulates HABR than portocaval shunting. More specifically, Doppler ultrasonography was used to obtain measurements of the hepatic artery and portal vein, with derived velocity parameters serving as indirect Indicators of alteration in vascular flow within these vessels.

## Materials and methods

We designed a prospective experimental animal cohort study using 10 female Landrace pigs weighing 25-30kg. The study was performed at the Experimental and Research unit of the Second Department of Surgery “Aretaieion” Hospital (National and Kapodistrian University of Athens, School of Medicine, Athens, Greece). The study protocol was approved by the Bioethics Committee of Aretaieion Hospital and the Animal Research Veterinary Committee of the prefecture of Athens, and was found in accordance with the National and European guidelines for ethical animal research and animal handling (regional reference number 524943/11-09-2019).

All 10 animals underwent surgery under sterile conditions and endotracheal intubation. After achieving general anesthesia, through a right lateral cervical incision, surgical exposure and dissection of the right internal carotid and internal jugular vein were performed for the insertion of arterial and venous catheters for invasive cardiovascular monitoring, fluid resuscitation, and medication administration. Then, a midline laparotomy was performed, and a urinary catheterization was made through a cystotomy. Subsequently, the infrahepatic inferior vena cava (IVC) and portal vein were mobilized. A side-to-side portocaval shunt (PCS) was constructed with a running 6-0 Prolene suture after partial occlusion of each vessel with Cooley clamps. The partial occlusion did not exceed 10 minutes to avoid intestinal venous congestion. A functioning PCS was necessary to avoid ischemic injury from venous congestion of the intestine during the subsequent hepatectomy. Baseline measurements of liver blood flow were made with ultrasound Doppler equipment. 

At this point, a left extended hepatectomy with Pringle maneuver was performed. Care was taken so the subjects would maintain hemodynamic stability throughout the hepatectomy. Subsequently, a stabilization period would follow where the PCS was temporarily occluded for the hemodynamic changes to take place, and that was confirmed through Doppler measurements. After the completion of the hepatectomy, the portocaval shunt was temporarily occluded, and repeated measurements were taken to establish the activation of the hepatic arterial buffer response. A sequential, within-subject interventional design was employed, whereby all animals were subjected to identical surgical and pharmacological procedures. This approach was chosen to reduce animal use in compliance with the 3Rs principles, as defined in Directive 2010/63/EU on the protection of animals used for scientific purposes, while preserving data quality and internal validity.

Initially, animals underwent the PCS maneuver with the shunt open to obtain initial measurements. This was followed by a 30-minute washout period with the shunt closed, during which all hemodynamic parameters returned to baseline. Subsequently, pharmacological intervention was carried out with the shunt closed via administration of 400 μg of regadenoson. Doppler measurement of the hepatic blood flow (both portal and arterial) was taken in intervals of 1 min, 5 min, and 60 min according to the half-life and interval time to maximal effect of regadenoson, and then the crossover was performed in the animals.

Measurements

All Doppler measurements were performed by a single operator at a standardised anatomical site within the vasculature to ensure consistency. Baseline measurements additionally included vessel diameter, enabling assessment of vasoconstriction and vasodilation. Velocity parameters were analysed in accordance with hemodynamic principles and served as indirect surrogates for changes in vascular flow. More specifically, for the purpose of this experiment, blood flow was indirectly estimated for any increase or decrease. We directly took measurements of the hepatic artery and portal vein velocities in centimeters per second (cm/s). According to the Bernoulli principle, during vasoconstriction, when a vessel’s diameter becomes smaller, the blood velocity increases, and conversely, it decreases during vasodilation [10]. Specifically, when the hepatic artery undergoes vasoconstriction, its systolic velocity typically rises. This phenomenon is explained by fundamental principles of fluid dynamics: to maintain continuity of flow through a narrowed segment, velocity must increase. In Doppler ultrasound, systolic velocity is a key parameter and is often elevated in regions of stenosis or vasoconstriction [11]. The relationship between hepatic artery velocity and blood flow is governed by the equation $\textit{Flow} = \textit{Velocity} \times \textit{Cross-sectional Area}$ [10-12,13]. ​​​While vasoconstriction increases velocity due to the reduced vessel diameter, the overall blood flow may remain unchanged or even decrease, depending on the extent of narrowing [10].

There were three main velocity measurements in the liver vasculature that were documented: a) estimated marginal means of portal vein velocity (PVV), b) estimated marginal means of hepatic artery systolic velocity (HASV), and c) estimated marginal means of hepatic artery diastolic velocity (HADV). These measurements were documented and analyzed in eight different time points, as listed in Table 1.

**Table 1:** Timeframe of the experimental measurements. PCS: portocaval shunt; HABR: hepatic arterial buffer response

Time Point	Description
Time 1	Before hepatectomy and before PCS
Time 2	After hepatectomy, closed PCS-HABR activated
Time 3	After hepatectomy, open PCS 1 min
Time 4	After hepatectomy, open PCS 5 min
Time 5	After hepatectomy, open PCS 60 min
Time 6	After hepatectomy, closed PCS, regadenoson administration 1 min
Time 7	After hepatectomy, closed PCS, regadenoson administration 5 min
Time 8	After hepatectomy, closed PCS, regadenoson administration 60 min

Statistical analysis

Variables were tested for normality using the Kolmogorov-Smirnov criterion. Quantitative variables are expressed as mean values (standard deviation). Repeated measures analysis of variance (ANOVA) was adopted to evaluate the changes observed in portal vein (PV) flow, HASV, and HADV values over the follow-up period. Sphericity assumption was checked via Mauchly’s test, and in cases where the assumption was not satisfied, the Greenhouse-Geisser assumption was applied. Bonferroni correction was used in the case of multiple testing in order to control for type I error. All reported p-values are two-tailed. Statistical significance was set at p<0.05, and analyses were conducted using SPSS statistical software (version 27.0; IBM Corp., Armonk, USA).

## Results

**Figure 1:**
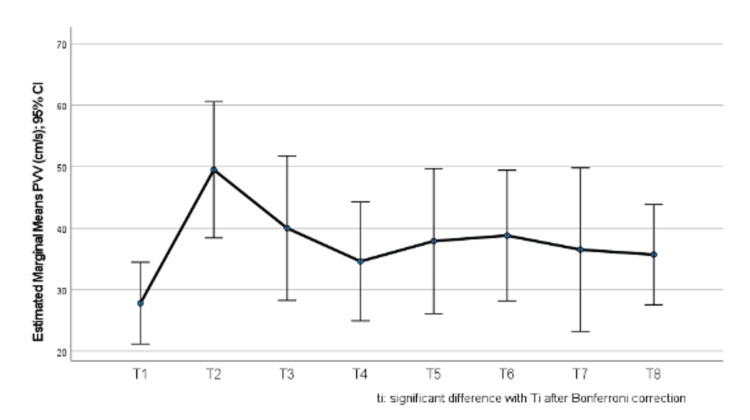
Change in portal vein velocity (PVV) throughout the study period T: time point

The sample consisted of 10 samples, measured at eight different time points each. Mean PV velocity values are presented in Table 2. There was a change in PV velocity values throughout the study period (p=0.019). After Bonferroni correction, it was found that at time point 2 (i.e., after hepatectomy/closed PCS/HABR), PV velocity values tended to be higher compared to time point 1 (i.e., baseline/before PCS); p=0.089 (Figure 1). Among the rest of the time points, no significant differences were found (p>0.05). 

**Table 2:** Portal vein velocity (PVV) per timepoint PCS: portocaval shunt; HABR: hepatic arterial buffer response; PV: portal vein

Time point		PV flow		
		Mean	SD	95% CI
T1.	Baseline/before PCS	27.8	9.3	21.1 ─ 34.5
T2.	After hepatectomy/closed PCS/HABR	49.5	15.5	38.4 ─ 60.6
T3.	After hepatectomy/open PCS 1 min	40.0	16.4	28.3 ─ 51.7
T4.	After hepatectomy/open PCS 5min	34.6	13.5	24.9 ─ 44.3
T5.	After hepatectomy/open PCS 1 h	37.9	16.5	26.1 ─ 49.7
T6.	After hepatectomy/closed PCS -regadenoson1min	38.8	14.9	28.2 ─ 49.4
T7.	After hepatectomy /closed PCS regadenoson 5 min	36.5	18.6	23.2 ─ 49.8
T8.	After hepatectomy/closed PCS regadenoson 1h	35.7	11.4	27.5 ─ 43.9
Change from T1 to T8		7.9	9.8	0.9 ─ 14.9
P-value for time effect		0.019		
Partial Eta squared		0.23		

**Figure 2:**
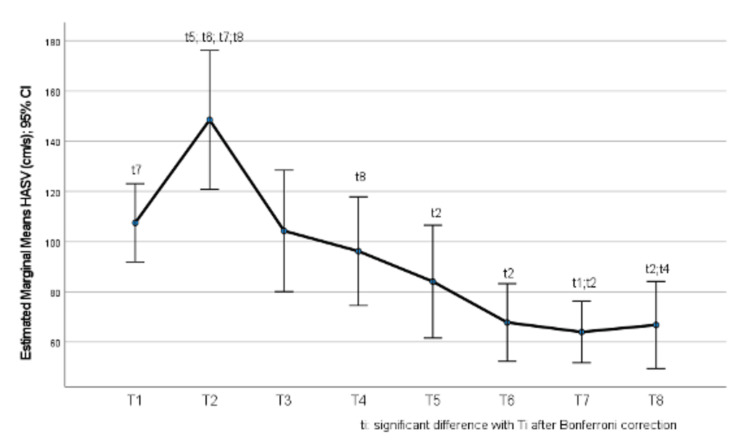
Change in hepatic artery systolic velocity (HASV) throughout the study period

Hepatic artery systolic velocity values by time point are presented in Table 3. There was a significant change in HASV values throughout the study period (p<0.001). More specifically, it was found that, after Bonferroni correction, at time point 2, HASV was significantly greater compared to time points 5 (p=0.013), 6 (p=0.001), 7 (p=0.005), and 8 (p<0.001), while it tended to be greater than that in time point 4 (p=0.066) (Figure 2). Also, at time point 4, HASV was significantly greater compared to time point 8 (p=0.042). Furthermore, at time point 1, HASV tended to be greater than at time point 7 (p=0.084). Among the remaining time points, no significant differences were found (p>0.05). 

**Table 3:** Hepatic artery systolic velocity (HASV) per timepoint PCS: portocaval shunt; HABR: hepatic arterial buffer response;

Time point		HASV		
		Mean	SD	95% CI
T1.	Baseline/before PCS	107.5	21.8	91.9 ─ 123.1
T2.	After hepatectomy/closed PCS/HABR	148.5	38.7	120.8 ─ 176.2
T3.	After hepatectomy/open PCS 1 min	104.3	33.8	80.1 ─ 128.5
T4.	After hepatectomy/open PCS 5 min	96.2	30.2	74.6 ─ 117.8
T5.	After hepatectomy/open PCS 1 h	84.1	31.4	61.6 ─ 106.6
T6.	After hepatectomy/closed PCS regadenoson 1 min	67.8	21.6	52.4 ─ 83.2
T7.	After hepatectomy/closed PCS regadenoson 5 min	64	17.2	51.7 ─ 76.3
T8.	After hepatectomy/closed PCS regadenoson 1h	66.8	24.3	49.4 ─ 84.2
Change from T1 to T8		-40.7	38.6	-68.4 ─ -13.1
P-value for time effect		<0.001		
Partial Eta squared		0.61		

**Figure 3:**
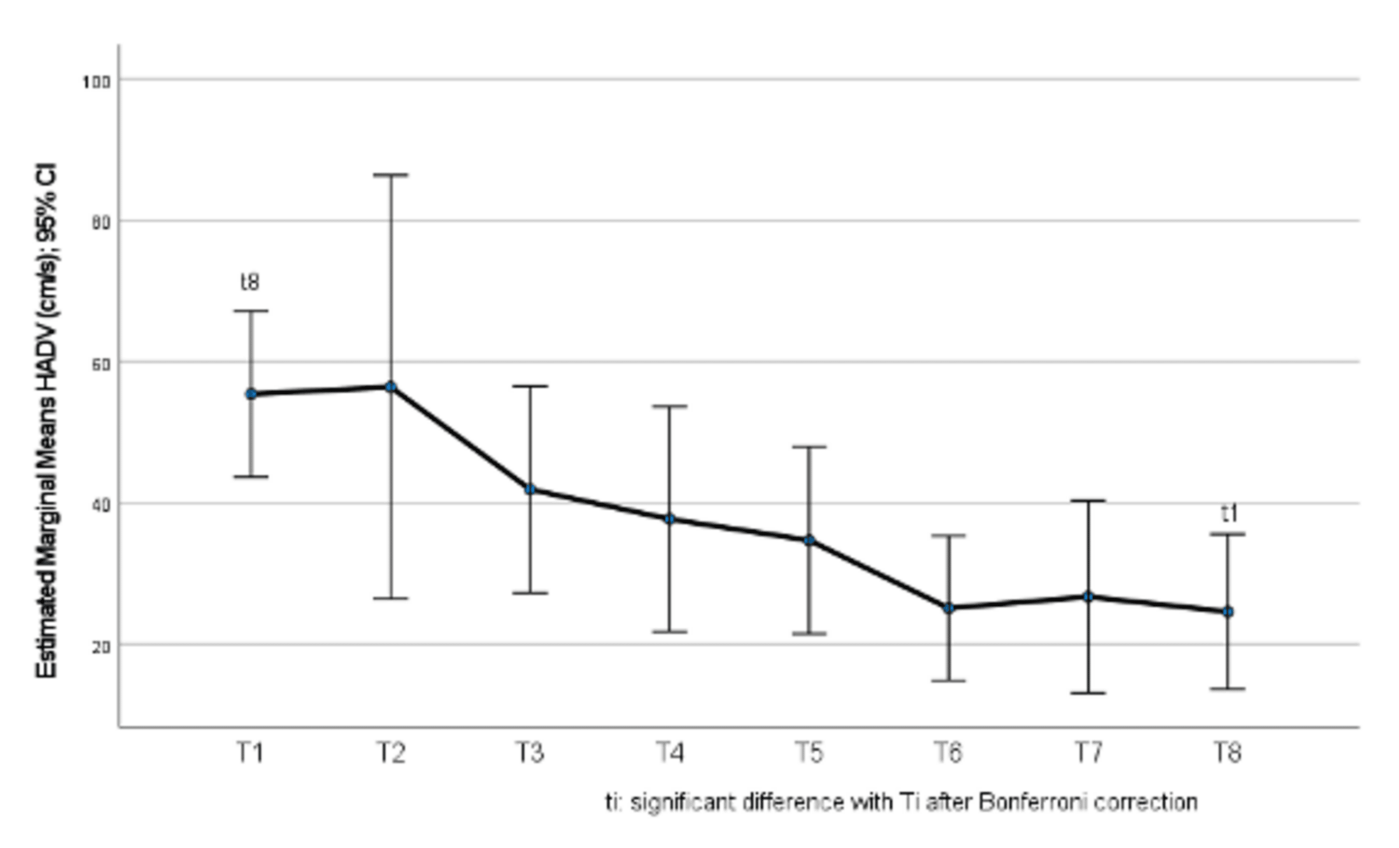
Change in hepatic artery diastolic velocity (HADV) throughout the study period

Hepatic artery diastolic velocity values are presented by time point in Table 4. There was a significant change in HADV values throughout the study period (p<0.001). More specifically, it was found that, after Bonferroni correction, the only significant difference was found between time points 1 and 8, when at time point 1 HADV was significantly greater compared to time point 8 (p=0.048) (Figure 3). Also, at time point 6, HADV tended to be lower than at time points 1 (p=0.088) and 3 (p=0.072). Among the rest of the timepoints, no significant differences were found (p>0.05).

**Table 4:** Hepatic artery diastolic velocity (HADV) per timepoint PCS: portocaval shunt; HABR: hepatic arterial buffer response

Timepoint		HADV		
		Mean	SD	95% CI
T1.	Baseline/before PCS	55.4	16.4	43.6 ─ 67.2
T2.	After hepatectomy/closed PCS/HABR	56.4	41.9	26.4 ─ 86.4
T3.	After hepatectomy/open PCS 1 min	41.9	20.4	27.3 ─ 56.5
T4.	After hepatectomy/open PCS 5 min	37.7	22.3	21.8 ─ 53.6
T5.	After hepatectomy/open PCS 1 h	34.7	18.5	21.5 ─ 47.9
T6.	After hepatectomy/closed PCS regadenoson 1 min	25.1	14.3	14.9 ─ 35.3
T7.	After hepatectomy/closed PCS regadenoson 5 min	26.7	19.0	13.1 ─ 40.3
T8.	After hepatectomy/closed PCS regadenoson 1h	24.6	15.3	13.6 ─ 35.6
Change from T1 to T8		-30.8	22.1	-46.6 ─ -15.0
P-value for time effect		<0.001		
Effect size		0.41		

In Figure 1, we can observe the estimated marginal means of portal vein velocity (PVV). There is an upward trend in the velocity of the portal vein between the baseline measurement T1 (before the hepatectomy) and the measurement of velocity at T2 (after the hepatectomy with a closed portocaval shunt). This could signify the timeframe of activation of HABR. 

In Figure 2, we can observe the estimated marginal means of systolic hepatic artery velocities. As we can observe, the highest velocity occurs at T2, where we observe the activation of the HABR phenomenon with the subsequent vasoconstriction of the hepatic artery. This actually proves the activation of this phenomenon. Additionally, there is a statistical significance in the velocities between the HABR activation (T2) and the reversal of this phenomenon by decreasing the velocities after the use of regadenoson and the portocaval shunt (T5, T6, T7, T8).

Portocaval shunting also decreases the velocities (T4), but to a lesser extent compared to regadenoson. So the hypothesis that it is more effective is proven with good statistical significance.

Finally, in Figure 3, we observe the estimated marginal means of the diastolic hepatic artery velocity. There is a statistical significance between the baseline measurement (T1) and the regadenoson administration (T8), with a proven statistical decrease.

## Discussion

Posthepatectomy liver failure remains a major complication in liver surgery, with HABR playing a key role in its pathophysiology. Defined by the International Study Group of Liver Surgery (ISGLS) as impaired synthetic and excretory liver function manifesting as elevated international normalized ratio (INR) and hyperbilirubinemia from postoperative day five onward, PHLF has various definitions globally, but ISGLS remains the most standardized [3]. Posthepatectomy liver failure is multifactorial, involving patient, surgery, and management-related risk factors [14].

While some factors like cirrhosis or patient age are non-modifiable, the most critical and challenging cause remains inadequate future liver remnant volume. This leads to an inability of the portal vein to regulate excessive flow, triggering HABR, a compensatory mechanism that reduces hepatic arterial inflow, depriving the regenerating liver of oxygen-rich blood. HABR is believed to be mediated by the "adenosine washout" hypothesis, whereby reduced portal flow results in adenosine accumulation in the space of Mall of the liver, leading to arterial vasodilation [15,16].

Adenosine directly induces hepatic artery dilation, and its effects are modulated by portal infusion, uptake agonists, and antagonists, supporting its central role in HABR regulation [15-18]. Systematically, portal hyperperfusion in a reduced liver can suppress hepatic arterial flow, rendering the liver functionally de-arterialized [19]. Interventions such as splenic artery ligation or embolization have shown promise in mitigating this effect, though these techniques carry procedural risks [20]. Given the regenerating liver’s high oxygen demand, pharmacologic strategies to enhance hepatic arterial flow are increasingly attractive [21,22].

Experimental studies have shown that intra-arterial adenosine improves hepatic artery flow and survival in small-for-size models [23]. Clinical investigations in Japan have similarly trialed direct drug infusion (e.g., prostaglandin E1) into portal circulation to mitigate injury [24]. Our study supports this by demonstrating that regadenoson, a selective A_2A_ receptor agonist, effectively reverses HABR, enhances hepatic arterial flow, and outperforms surgical portocaval shunting in preventing PHLF. Surgical shunts may alleviate portal hyperperfusion and reverse HABR [15,16,25,26], but their clinical use is limited by complications like hepatic encephalopathy [27]. In contrast, regadenoson has shown favorable effects in liver transplant models by improving perfusion, reducing histological injury, and modulating inflammation via A2A receptor activation [17,23,28]. Regadenoson’s selectivity for A_2A_ receptors (K_i_ ≈ 1.3 μM) and minimal off-target effects (A_1_ (Ki > 16.5 μM), A_2B_/A_3_) make it a compelling therapeutic option. Already used in cardiac stress testing, its splanchnic vasodilatory properties suggest a novel application in managing PHLF, as shown in our experimental findings.

This experimental study evaluated the dynamic alterations in hepatic hemodynamics following left extended hepatectomy, portocaval shunt modulation, and selective pharmacologic vasodilation using regadenoson in a porcine model. The findings support the functional activation of the HABR post-hepatectomy and reveal in detail measurable hepatic flow effects associated with regadenoson administration.

Portal vein velocity demonstrated an overall change throughout the study period (p = 0.019), with a trend toward increased values immediately after hepatectomy in the presence of a closed portocaval shunt (time point 2), compared to baseline (p = 0.089). This observation is consistent with HABR physiology, where an increase in portal perfusion induces compensatory hepatic arterial vasoconstriction that subsequently leads to PHLF [4,5,15,16,19]. This difference reaches statistical significance after Bonferroni correction, a trend that aligns with established experimental data on intrahepatic hemodynamic regulation [5,15]. HASV also varied significantly across timepoints (p < 0.001), with the highest values observed shortly after hepatectomy (time point 2), followed by a progressive decline, particularly after regadenoson administration (time points 6 to 8). These findings likely reflect both the initial surgical stress response and the systemic vasodilatory effects of regadenoson, a selective adenosine A2A receptor agonist.

The drug's known mechanism - vasodilation through activation of endothelial nitric oxide and smooth muscle relaxation - explains the observed reductions in HASV and, to a lesser extent, diastolic velocities (HADV), which also showed a statistically significant decrease between time points 1 and 8 (p = 0.048). The hemodynamic changes observed following regadenoson administration emphasize its potential role in modulating hepatic perfusion pharmacologically. In clinical contexts, this may have implications for enhancing liver perfusion post-resection or during ischemia-reperfusion scenarios. However, the systemic hypotensive effect also necessitates caution, particularly in patients with marginal cardiovascular reserve.

Despite these important findings, the study has several limitations. The small sample size may limit the statistical power to detect more subtle hemodynamic differences, and the acute nature of the experimental model does not account for longer-term adaptations. Additionally, Doppler-derived velocity measurements, while widely accepted, may not precisely reflect volumetric flow without concurrent assessment of vessel diameter. Furthermore, although the use of an animal model is physiologically relevant, it does not fully replicate human clinical conditions. Therefore, further studies incorporating direct flow quantification, tissue oxygenation markers, and long-term outcomes, as well as clinical investigations in human subjects, are warranted to validate these findings, confirm efficacy, and better define optimal therapeutic protocols.

## Conclusions

This study underscores the physiological presence and significance of HABR following extended hepatectomy. Our findings demonstrate that both surgical (portocaval shunt) and pharmacological (intra-arterial regadenoson) interventions can effectively modulate this response. Notably, regadenoson significantly enhanced hepatic arterial perfusion and could potentially reduce the incidence of PHLF compared to surgical modulation alone in the porcine model. These results position regadenoson as a promising therapeutic agent for optimizing liver perfusion, particularly in cases where portal venous flow is impaired. The superior outcomes associated with pharmacological intervention highlight the potential for targeted therapies to support hepatic hemodynamics in major liver resections. Further clinical studies are warranted to validate these findings and explore their translational impact in human liver surgery.
